# The use of a novel complication reporting system: the comprehensive complication index in thulium laser enucleation of the prostate

**DOI:** 10.1186/s40001-025-03052-x

**Published:** 2025-08-19

**Authors:** Song Haifeng, Daxun Luo, Hui Liu, Yunfei Fan, Haiwen Huang, Yubao Liu, Meng Fu, Jianxing Li

**Affiliations:** Department of Urology, Beijing Tsinghua Changgung Hospital, School of Clinical Medicine, Tsinghua University, Beijing, 102218 China

**Keywords:** Benign prostate hyperplasia, Thulium laser enucleation, Comprehensive complication index, Clavien–Dindo classification

## Abstract

**Objectives:**

To evaluate the application of the comprehensive complication index (CCI) in the assessment of postoperative complications after thulium laser prostate enucleation (ThuLEP) and compare it with the Clavien‒Dindo classification (CDC) grading system.

**Methods:**

A retrospective analysis of the clinical data of 467 patients hospitalized for benign prostatic hyperplasia treated with ThuLEP surgery from August 2019 to October 2023 was conducted. The postoperative complications of patients were assessed via both the CDC and CCI complication evaluation systems, and the correlation between both systems and the postoperative length of stay (LOS) was analyzed. Sample size estimations for randomized controlled trials (RCTs) were also performed on the basis of complication rates and CCI scores.

**Results:**

Ninety-nine (21.20%) of the patients had a total of 121 complications. Eighteen patients experienced ≥ 2 types of complications, and their cumulative CCI scores exceeded the score corresponding to the highest CDC grade. Both the CDC and CCI were significantly and positively correlated with the postoperative LOS, with the cumulative CCI score showing a stronger correlation than the highest CDC grade (*r* = 0.429, *p* < 0.001 vs. *r* = 0.383, *p* < 0.001). Furthermore, the use of the CCI for evaluating postoperative complications was found to reduce the required sample size for RCTs (305 per group vs. 90 per group).

**Conclusions:**

Compared with the CDC, the CCI more accurately reflects short-term postoperative complications after ThuLEP and shows a stronger correlation with LOS while enabling smaller sample sizes in RCTs. Future studies should integrate the CCI into standardized reporting for laser-based BPH surgery to improve consistency and benchmarking.

## Introduction

With the advancements in minimally invasive techniques and laser medicine, laser enucleation of the prostate is gradually replacing traditional transurethral resection of the prostate (TURP) as the new standard for the surgical treatment of benign prostatic hyperplasia (BPH) [1]. Despite continuous improvements in surgical methods, the assessment of postoperative complications remains crucial. Currently, the Clavien‒Dindo classification (CDC) system is the most widely used system for evaluating surgical complications [2]. The application of this system has been recommended for BPH surgeries, including TURP, holmium laser enucleation of the prostate (HoLEP) and thulium laser enucleation of the prostate (ThuLEP) [3, 4]. However, the CDC system has significant limitations and shortcomings [5].

The comprehensive complication index (CCI) is a novel indicator for evaluating postoperative complications, and its value has been recognized and emphasized in various surgeries[6–9]. Reports suggest that, compared with the CDC, the CCI offers greater sensitivity and clinical applicability and can reduce the sample size required for randomized controlled trials (RCTs) [10]. However, few studies have evaluated the use of the CCI in assessing postoperative complications in ThuLEP patients at present, and its application in this context needs further evaluation. Therefore, this study aimed to report and evaluate postoperative complications associated with the CCI in ThuLEP patients and compare them with those associated with the CDC.

## Methods

### Patient population surgical procedure

We retrospectively collected clinical data from 467 patients who underwent ThuLEP surgery for benign prostatic hyperplasia (BPH) at the Department of Urology, Beijing Tsinghua Changgung Hospital, between August 2019 and October 2023.

Inclusion criteria were: men undergoing primary ThuLEP for symptomatic BPH with the three-lobe technique; availability of complete perioperative records; index admission length of stay ≥ 24 h.

Exclusion criteria were: prior prostate enucleation; concomitant other endoscopic procedures during the same session (e.g., TURBT, ureteral stone intervention); suspected prostate cancer; active urinary tract infection at the time of surgery; missing perioperative data.

### Surgical procedure

All patients underwent ThuLEP via the three-lobe technique. First, a U-shaped incision was made approximately 5 mm from the verumontanum to expose the surgical capsule. Next, early apical urethral mucosa release was performed, followed by expansion of the plane to the 3 o'clock and 9 o'clock positions on each side. An incision was then made at the 12 o'clock position to perform an anterior commissurotomy. Two incisions were subsequently made from the bladder neck in an anterograde direction at the 5 o'clock and 7 o'clock positions. This was followed by retrograde enucleation of the median lobe. Then, the left and right lobes were enucleated. Finally, the enucleated prostatic tissue was removed via mechanical morcellation.

### Data collection

There were no cases of mortality. The collected data included age, body mass index (BMI), American Society of Anesthesiologists (ASA) score, prostate volume, postvoid residual (PVR) volume, International Prostate Symptom Score (IPSS), operation time, and postoperative length of stay (LOS).

### Assessment of complications

Postoperative complications occurring during the index hospitalization only were recorded through the electronic medical records system, utilizing both clinical notes and physician orders. Post-discharge complications (e.g., within 30 or 90 days) were not systematically captured in this retrospective data set. Each complication was graded individually according to the Clavien–Dindo classification (CDC) system.

The CCI score is calculated by assigning different weights to each CDC grade and then aggregating all complications into a single score, ranging from 0 (no complications) to 100 (death). The formula for the CCI is as follows: $CCI=\frac{\sqrt{wC1+wC2+\dots +wCx}}{2}$, where wC represents the weight corresponding to the CDC grade. The weights assigned to each CDC grade and their corresponding CCI scores are shown in Table 1. An online CCI calculator (www.assesssurgery.com) was used to calculate the cumulative CCI score for all postoperative complications of each patient. The correlations among the highest CDC grade, cumulative CCI score, and postoperative LOS were analyzed.

**Table 1:** Weights and corresponding CCI scores for different CDC grades

CDC grade	wC	CCI score
0	0	0
I	300	8.7
II	1750	20.9
IIIa	2750	26.2
IIIb	4550	33.7
IVa	7200	42.4
IVb	8550	46.2
V	NA	100

### Statistical analysis

The Kolmogorov‒Smirnov test was used to assess the normality of the data. Continuous variables that followed a normal distribution are presented as the mean ± standard deviation (SD) and were compared between groups via Student's *t* test. Nonnormally distributed continuous variables are presented as medians and interquartile ranges (IQRs), with group comparisons conducted via the nonparametric Mann‒Whitney *U* test. Categorical variables were compared via the chi-square test. Correlation analysis was performed via Spearman's rank correlation coefficient. The sample size for a hypothetical future superiority trial was estimated under the assumption of a 40% reduction in the occurrence of complications and a 2.94-point reduction in the CCI score, which was deemed clinically relevant owing to the lower overall complication rate. The assumed between-group difference (Δ) of 2.94 was based on the observed mean CCI in our cohort. This corresponds to approximately one-third of a low-grade complication per patient on average, which is clinically plausible and achievable in practice. With the cohort-specific SD of 6.98, this yields a standardized effect size (Cohen’s d) of ≈0.42, indicating a small-to-moderate effect. The 40% reduction in complication incidence (CDC) was based on a clinically relevant improvement threshold commonly used in prior randomized trials evaluating perioperative outcomes[5, 10]. The sample size calculation was conducted with a significance level of *α* = 0.05 and a power of 1-β = 0.8. Data analysis and visualization were performed via MedCalc (version 19.1.3; MedCalc Software Ltd., Ostend, Belgium) and R (version 4.0.2).

## Results

The median age of the 467 patients was 70 (65–76) years, with a BMI of 24.61 ± 3.67. Among them, 99 patients experienced postoperative complications. Patients were divided into two groups according to the presence or absence of postoperative complications. The baseline characteristics of the two groups are compared in Table 2. Compared with the group without complications, the group with complications had a significantly longer postoperative LOS [5 [4–6] vs. 3 (2–4) days, *p* < 0.001]. There were no significant differences between the two groups in terms of BMI, ASA score, prostate volume, PVR, IPSS or operation time.

**Table 2:** Baseline characteristics of 467 patients undergoing ThuLEP

Variables	Total (467)	With Complications (*n* = 99)	Without Complications (*n* = 368)	*p* value
Age (years),	70(65–76)	71(66–78)	70(65–76)	0.188
BMI (kg/m^2^)	24.61 ± 3.67	24.35 ± 3.61	24.67 ± 3.43	0.420
ASA				
≤ 2	230	46(20.00%)	184(80.00%)	0.533
> 2	237	53(22.40%)	184(77.60%)	
Prostate Volume(ml)	50.65(32.49–78.05)	56.53(35.86–78.61)	48.93(31.894–77.47)	0.285
PVR (ml)	114.00(50.00–212.00)	101.50(49.50–215.50)	116.50(50.00–210.00)	0.808
IPSS Score	21(19–25)	22(19–26)	21(20–25)	0.457
Operation time (minutes)	79.00(61.00–110.00)	77.50(60.00–112.00)	79.00(62.00–110.00)	0.886
Postoperative LOS (day)	4(2–5)	5(4–6)	3(2–4)	** < 0.001**

Among the 467 patients, 99 patients (21.20%) experienced a total of 121 complications. According to the CDC, there are 86 Grade I complications (18.42%), 25 Grade II complications (5.35%), 6 Grade IIIa complications (1.28%), 3 Grade IIIb complications (0.64%), and 1 Grade IVa complication (0.21%). No Grade IVb or Grade V complications were observed.

The most common Grade I and II complications were bladder spasms and constipation. The grade III complications included 5 cases of bladder tamponade, 1 case of acute coronary syndrome, 2 cases of urinary retention following catheter removal, and 1 case that required radical prostatectomy due to postoperative pathology indicating prostate cancer. The Grade IVa complication involved a patient who developed new-onset atrial fibrillation with rapid ventricular response and acute heart failure, requiring treatment in the intensive care unit.

**Fig. 1:**
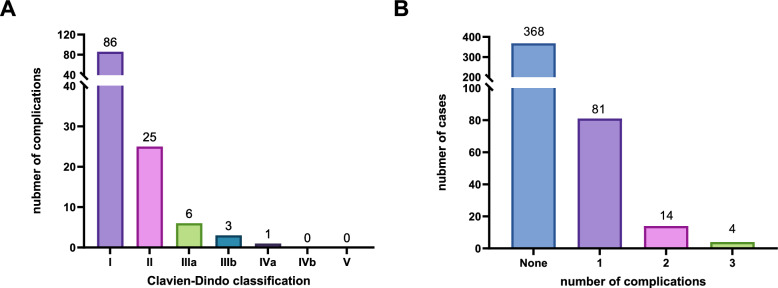
Postoperative complications following ThuLEP.** A** Distribution of postoperative complications according to the CDC grade among 467 patients. **B** Number of postoperative complications per patient among the 467 patients

There were 7 cases of infection, and 6 patients required blood transfusions, resulting in a transfusion rate of 1.06%. Among the 99 patients with complications, 81 (81.82%) experienced one type of complication, 14 (14.14%) experienced two types, and 4 (4.04%) experienced three types. The distribution of postoperative complications is illustrated in Fig. 1.

**Fig. 2:**
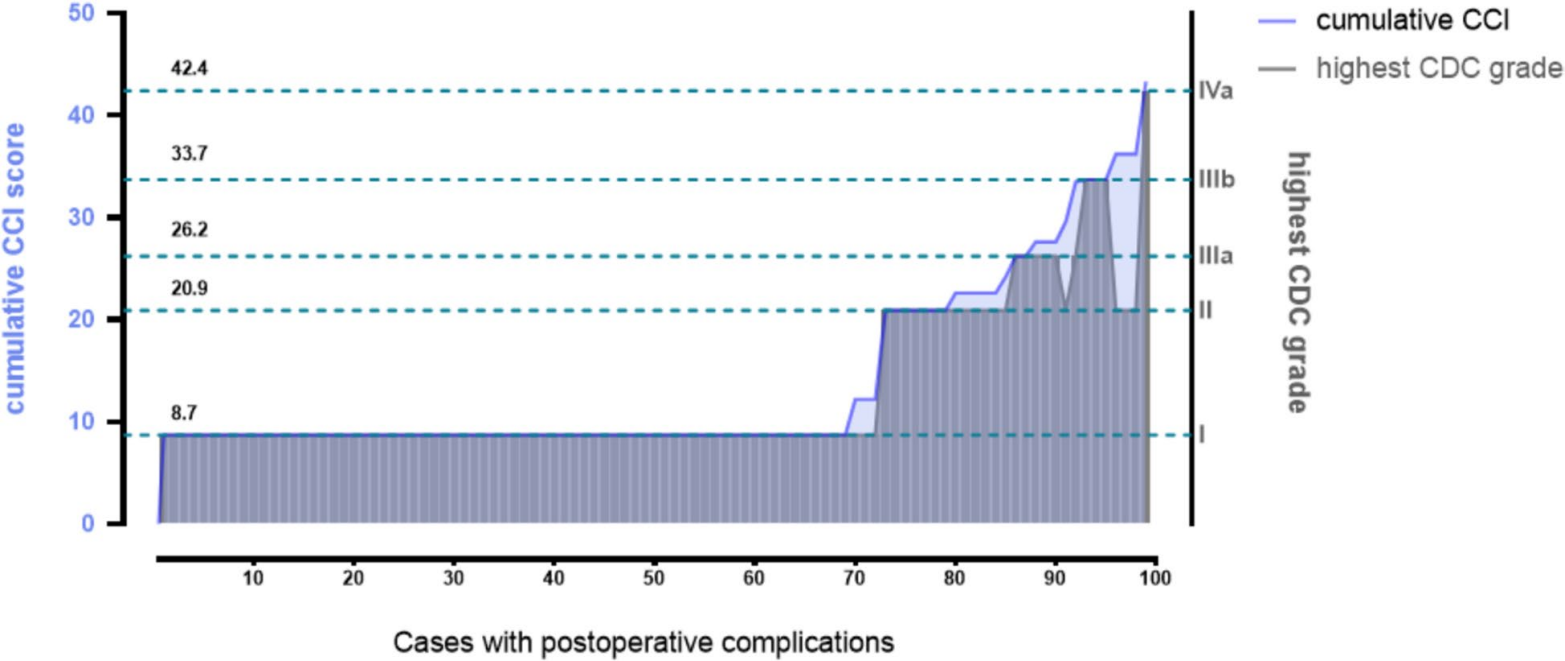
Comparison of the CDC and CCI in the evaluation of postoperative complications following ThuLEP. The gray bars represent the highest CDC grade for each patient. The blue curve shows the cumulative CCI scores for the corresponding patients

Among patients with complications, the mean cumulative CCI was 13.94 ± 9.14. For the 18 patients who experienced two or more complications, the cumulative CCI scores exceeded the CCI values corresponding to their highest CDC grade (Fig. 2). These findings indicate that the cumulative CCI is more representative of the burden of postoperative complications than the CDC score is.

**Fig. 3:**
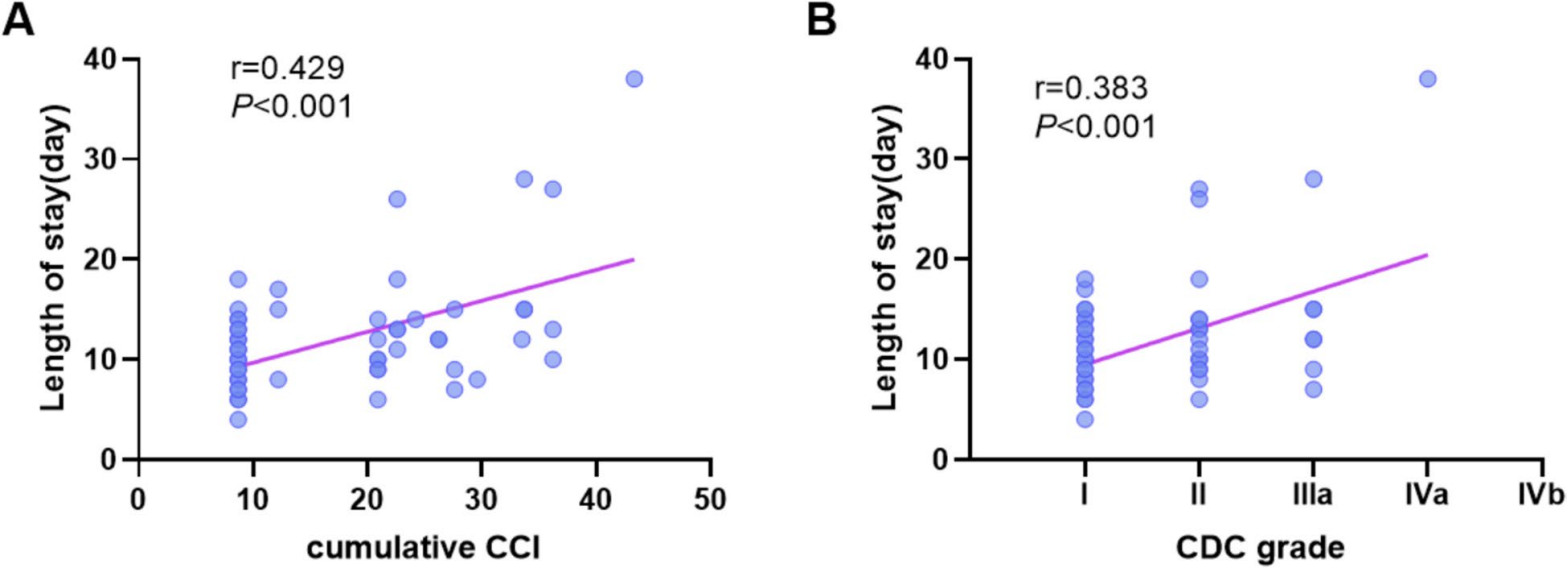
Correlations between the cumulative CCI score, highest CDC grade, and postoperative LOS. **A** Correlation analysis between the cumulative CCI score and postoperative hospital stay. **B** Correlation analysis between the highest CDC grade and postoperative hospital stay

The correlation analysis between the cumulative CCI score, highest CDC grade, and postoperative LOS revealed that both metrics were significantly positively correlated with the length of postoperative hospital stay. Specifically, higher CDC grades were associated with longer postoperative hospital stays (Fig. 3A), and higher cumulative CCI scores were also correlated with extended hospital stays (Fig. 3B). However, the cumulative CCI demonstrated a stronger correlation with postoperative hospital stay than did the highest CDC grade (*r* = 0.429, *p* < 0.001 vs. *r* = 0.383, *p* < 0.001).

In RCTs evaluating complications, a sufficient sample size is needed to detect statistically significant differences between groups. Based on the data from this study, we assumed the following differences: a 40% reduction in the incidence of complications when the CDC classification was used and a difference of 2.94 points (with a standard deviation of 6.98) in the CCI between groups. The estimated sample sizes for these scenarios are shown in Table 3. The CCI significantly reduced the required sample size for the study (305 per group vs. 90 per group).

**Table 3:** Estimated sample size for simulated clinical trials using different complication evaluation methods

Method		Assumption	Sample size (per group)
CDC grade	99/467 = 21.20%	40% reduction in complication	305
CCI	2.94 ± 6.98	Δ = 2.94, SD = 6.98	90

## Discussion

BPH is one of the most common diseases that significantly affects the quality of life of elderly men. The primary clinical manifestations include frequent urination, urgency, nocturia, and difficulty urinating, which are collectively known as lower urinary tract symptoms (LUTS). Reports indicate that the prevalence of BPH is 20% in men over 40 years of age, 50% in men over 60 years of age, and 83% in men over 80 years of age (11). For patients with moderate to severe LUTS that severely impact quality of life and are unresponsive to medical treatment or when complications arise, surgical intervention becomes necessary[12].

In recent years, transurethral laser surgery has become a key method for treating BPH. Compared with traditional transurethral procedures such as TURP, laser surgery offers advantages such as reduced bleeding and the absence of transurethral resection syndrome [13]. Compared with HoLEP and traditional TURP, ThuLEP offers similar surgical efficacy but results in less bleeding, faster recovery and a lower incidence of short-term postoperative urinary incontinence [14, 15].

The CDC grading system, recommended by the European Association of Urology (EAU), is widely used for reporting postoperative complications in transurethral surgeries [16, 17]. While the CDC is simple, intuitive, and easy to standardize, it has notable limitations. The system classifies complications based on the type of intervention required rather than the severity or mortality risk. In addition, studies have shown that in patients with multiple complications, only the highest grade complication is usually recorded, leading to the omission of lower grade complications. Moreover, as a categorical variable, the CDC cannot adequately compare the impact of different complication grades, such as two Grade II complications versus one Grade III complication [18].

The CCI was introduced by Slankamenac et al. in 2013 to address the shortcomings above [6]. Unlike the CDC, the CCI accounts for all postoperative adverse events, assigning weighted values and transforming them into a continuous variable ranging from 0 (no complications) to 100 (death). Initially, developed for general surgeries, such as gastric [7, 19], pancreatic [8, 20], colorectal [21], and liver surgeries [9, 22], the CCI has demonstrated greater sensitivity and clinical utility.

In recent years, the application of the CCI in the field of urology has been increasingly reported. Vetterlein et al. [23] and Haas et al. [24] documented the use of the CCI in evaluating postoperative complications following radical cystectomy for bladder cancer. Grüne B et al. reported the use of the CCI for assessing complications in patients undergoing percutaneous nephrolithotomy and ureteroscopy for urinary stones [25]. Geiger S et al. [26] further explored the application of the CCI in evaluating complications after nephroureterectomy. These studies suggest that CCI offers unique advantages in the field of urology as well. With respect to transurethral surgeries of the lower urinary tract, Waldbillig et al. reported that the CCI provides a more accurate assessment of postoperative complications for procedures, such as TURP, transurethral resection of bladder tumors (TURBT), and ThuLEP. In addition, using the CCI can reduce the sample size required for clinical trials, thereby alleviating the challenges associated with participant recruitment [5].

In this study, the overall incidence of complications was 21.20%, with Grade I complications (86 cases, 18.42%) and Grade II complications (25 cases, 5.35%) being the most common. This finding is consistent with reports from other studies [5]. The mean CCI for patients who experienced postoperative complications was 13.94 ± 9.14, indicating that most complications following ThuLEP are relatively minor, which reflects the safety of the ThuLEP procedure. Notably, 18 patients experienced two or more complications, with their cumulative CCI exceeding the CCI corresponding to their highest CDC grade. This suggests that relying solely on the CDC classification may underestimate the impact of complications in these cases. With respect to the prediction of the postoperative hospital stay, both the CDC and CCI were significantly correlated with the postoperative LOS. However, the correlation between the cumulative CCI and postoperative hospital stay was stronger than that between the highest CDC grade and hospital stay, indicating that the CCI has a greater impact on hospital stay. In addition, this study demonstrated that the use of the CCI to assess postoperative complications requires a smaller sample size in RCTs than the use of the CDC does, which aligns with previous research findings [10, 25, 27].

BPH is one of the most common diseases of the urinary system, and the number of patients undergoing surgical treatment for BPH continues to rise. Thorough assessment of postoperative complications is, therefore, of great clinical importance. The introduction of the CCI helps quantify and improve the accuracy of these assessments. This study is the first to focus on evaluating the application of the CCI in assessing complications following ThuLEP and to systematically compare the correlations among the CCI, CDC, and postoperative hospital stay. These findings indicate that the CCI is strongly correlated with the postoperative LOS, which may enhance the ability to predict patient recovery trajectories and optimize hospital management strategies. Furthermore, by simulating sample size calculations using both the CCI and the traditional CDC, it was found that, as a continuous, patient-level morbidity metric, CCI can reduce required sample sizes and enhance sensitivity to detect clinically meaningful differences—particularly in clinical trials, where complications are predominantly low-grade yet multiple. For ThuLEP and related endoscopic techniques—where severe events are uncommon—the CCI captures the cumulative burden more completely than traditional binary CDC endpoints. This facilitates feasible, cost-efficient trials, improves comparability across centers, and may accelerate the evaluation of perioperative optimization strategies.

This study has several limitations. First, as a single-center retrospective study, the assessment of postoperative complications was primarily based on postoperative orders and medical records, with no outpatient follow-up information. This may introduce information bias and could lead to an underestimation of the number of complications. Second, the sample size of this study was relatively small. Given that postoperative complications following ThuLEP are generally infrequent and mostly mild, the small sample size may result in a disproportionate influence of a few high-CCI cases on the overall study results. Finally, the CCI itself has limitations. Since the CCI was developed on the basis of the CDC, it similarly does not account for intraoperative complications. In addition, the intervention measures taken for the same complication may vary depending on the physician or center preference, which could lead to differences in final grading and scoring. Therefore, further large-scale, multicenter, prospective studies are needed to validate the applicability of the CCI in the field of urology.

## Conclusion

The CCI provides an accurate evaluation and reporting of postoperative complications following ThuLEP. Compared with the CDC, the CCI was more strongly correlated with the postoperative LOS. In addition, using the CCI can reduce the sample size required for RCTs of ThuLEP. Future studies should integrate the CCI into standardized perioperative complication reporting protocols for laser-based BPH surgery to improve consistency in outcome reporting and benchmarking across institutions.

## Data Availability

No datasets were generated or analysed during the current study.
